# Trogocytic intercellular membrane exchanges among hematological tumors

**DOI:** 10.1186/s13045-015-0114-8

**Published:** 2015-03-14

**Authors:** Joel LeMaoult, Julien Caumartin, Marina Daouya, Magdalena Switala, Vera Rebmann, Bertrand Arnulf, Edgardo D Carosella

**Affiliations:** CEA, Institute of Emerging Diseases and Innovative Therapies (iMETI), Research Division in Hematology and Immunology (SRHI), Saint-Louis Hospital, Paris, France; University Paris Diderot, Sorbonne Paris Cité, UMR E_5 Institut Universitaire d’Hematologie, Saint-Louis Hospital, Paris, France; Biology and Biotechnology Ph.D. Program, Univ Paris Diderot, Sorbonne Paris Cite, Paris, France; Institute for Transfusion Medicine, University Hospital Essen, Essen, Germany; Département d’Immuno-Hématologie, Hôpital Saint-Louis, Paris, France

**Keywords:** Trogocytosis, Leukemia, HLA-G, Tumor escape, Immune regulation

## Abstract

Trogocytosis is the transfer of plasma membrane fragments and the molecules they contain between one donor and one acceptor/acquirer cell. Through trogocytosis, acceptor cells temporarily display and use cell-surface molecules they do not express themselves, but borrow from other cells. Here, we investigated whether liquid tumors possessed a trogocytic capability, if immune escape molecules could be acquired by tumor cells, transferred between cells of the same tumor, and if this could benefit the tumor as a whole.

For this, we investigated trogocytosis in hematological cell lines and freshly isolated hematological tumor cells. We demonstrate that hematological tumor lines possess a trogocytic capability that allows them to capture membranes that contain the immune-inhibitory molecule HLA-G from allogeneic as well as from autologous sources. We further show that freshly isolated hematological tumor cells also possess these capabilities. This work reports for the first time the trogocytic capabilities of liquid tumor cells and introduces the notion of immune escape strategy sharing among tumor cells through trogocytosis of membrane-bound immune-inhibitory molecules.

**Electronic supplementary material:**

The online version of this article (doi:10.1186/s13045-015-0114-8) contains supplementary material, which is available to authorized users.

## Introduction

HLA-G is a tolerogenic molecule which expression was originally observed and characterized on throphoblasts. Even though it has recently been suggested that it participates in the induction of trophoblast cell fusion [1,2], HLA-G is best known for its ability to confer protection to the semi-allogeneic fetus from the maternal immune system [3,4]. HLA-G differs from classical MHC class I molecules by its genetic diversity, expression, structure, and functions. It is characterized by a relatively low allelic polymorphism and a highly restricted tissue distribution. HLA-G constitutive expression is mainly restricted to trophoblast cells [3], and to adult thymic medulla [5], pancreatic islets [6], and stem cells [7,8]. However, HLA-G can be neo-expressed in pathological conditions such as transplantation [9], inflammatory and autoimmune diseases [10], viral infections [11], and cancers [12]. HLA-G expression is under the control of epigenetic mechanisms. In most adult tissues, HLA-G gene expression in repressed by methylation, which can be reversed by demethylating agents such as 5-aza-2′-deoxycytidine [13]. Of note: such demethylating agents are used in cancer therapy; they might therefore have the adverse effect of inducing immune escape through HLA-G expression. Concerning HLA-G expression regulation, it is under the control of polymorphism and microenvironmental factors such as hypoxia and cytokines, and miRNA such as MiR148a and MiR152 (for review, see [14,15]).

HLA-G is exclusively immune-inhibitory. Under its membrane-bound and soluble forms, it is able to inhibit NK cells and cytotoxic T lymphocyte cytolytic activity [4,16-19], proliferative T cell responses, T cell and NK cell ongoing proliferation [20-22], and dendritic cell maturation [23,24]. Recent studies have also shown that HLA-G is capable of inducing the differentiation of regulatory T cells and antigen-presenting cells (APCs), which can then inhibit immune responses themselves [20,23,25-27].

HLA-G neo-expression has been detected in several human cancers including melanoma, renal cell carcinoma, breast carcinoma, and large cell carcinoma of the lung [12,28-32]. HLA-G expression by tumor cells has been shown to be important for the escape of immune surveillance by host T lymphocytes and NK cells [12,28,29,33-35]. HLA-G promoter specificities even allow its expression when that of classical HLA-class-I molecules is downregulated. This is particularly evident in trophoblast cells, but also occurs in tumor cells. Thus, HLA-G expression by malignant cells may prevent tumor immune eradication by inhibiting the activity of tumor infiltrating NK, cytotoxic T lymphocytes (CTL), and APCs. The clinical relevance of HLA-G expression by tumors as a prominent immune escape mechanism was supported by numerous studies and for both solid and liquid tumors (for review, see [36,37]). HLA-G is not a tumorigenic molecule per se, but it could contribute to tumorigenesis if expressed by cancer stem cells or precancerous cells, shielding them from immune destruction during their evolution processes. Of particular relevance to our study, HLA-G expression in B cell chronic leukemia correlated with a strong immunodeficiency and poor clinical evolution [38,39].

HLA-G acts mainly through two inhibitory receptors: LILRB1/ILT2 and LILRB2/ILT4 that are differentially expressed by NK, T, and B cells, and myeloid APCs. HLA-G was also reported to exert its tumor immune escape functions by the mechanism of trogocytosis [22,40].

Trogocytosis is a mechanism of rapid transfer of membranes and membrane-associated proteins between interacting cells. Membrane transfers have been observed mainly between immune cells [41], and in particular for HLA molecules. Although trogocytic transfers are common and rather easy to observe experimentally, the mechanisms that underlie them and the molecules directly responsible for transfer remain unclear. In particular, trogocytosis was shown to be dependent on MHC-TCR interactions or costimulatory molecules (for review, see [42]). Our laboratory described the trogocytic transfers of HLA-G and its receptor ILT2 [22,40,43]. In these studies, we could not identify which molecules were responsible for APC-to-T or tumor-to-NK, or tumor-to-APC trogocytosis. However, we did demonstrate that in APC-to-T trogocytosis, multiple molecules transferred to various degrees. Among these, MHC molecules and CD86 transferred the most, a finding that is compatible with the transfer of membrane-bound organized molecular clusters such as immune synapses or lipid rafts [40,44]. After trogocytic acquisition, the transferred molecules retain their original function and it is now well established that trogocytosis of the HLA-G molecule can impact the outcome of an immune reaction by conferring protection to the HLA-G acceptor cell, and by conferring sensitivity to inhibition by HLA-G through the transfer of its receptor ILT2 [22,40,43,45,46]. Most of these studies have been performed using immune cells as membrane acceptor cells (T cells, B cells, monocytes), and tumor cell lines as membrane-donor cells [22,40,46,47]. In two studies [22,48], we showed that the NK cell line NKL was able to efficiently acquire HLA-G1-containing membranes from an allogeneic HLA-G1-expressing melanoma line through trogocytosis [22]. These experiments demonstrated that hematological tumor cells may also be trogocytic, although it cannot be assumed that tumor-to-tumor transfers occur by the same mechanisms as APC-to-T cell or tumor-to-effector transfers. Other reports showed that trogocytosis occurred between autologous cells [43,46-48]. Taken together, these data suggest that tumor cells may exchange membranes and proteins by trogocytosis among each other.

In the present report, we demonstrate *in vitro* and *ex vivo* that tumor cell lines of immune origin, and tumor cells from malignant hemopathies such as lymphoma or leukemia malignancies, possess trogocytic capabilities: they can acquire membranes and the membrane-bound immune escape molecule HLA-G1 from their surroundings and from each other.

## Materials and methods

### Cells and cell lines

Blood was obtained from patients after informed consent according to the Declaration of Helsinki under protocol approved by the Institutional Review Board of the St Louis Hospital, Paris, and participants provided their written informed consent to participate in this study. Samples were processed and treated anonymously. The cell lines used in this study were of monocytic origin: histiocytic lymphoma (monocyte) U937 cells, acute monocytic leukemia THP-1 cells, HL-60, and promyelomonocytic leukemia KG-1 cells; B cell origin: lymphoblastoid LCL721.221 cells, Burkitt’s lymphoma Raji cells, Burkitt’s lymphoma Ramos cells, myeloma RPMI8226 cells, and myeloma U266 cells; T cell origin: acute T cell leukemia Jurkat cells; and NK cell origin: NK leukemia NKL cells. LCL721.221 cells transfected with the HLA-G1 cDNA (LCL-HLA-G1) have been described [49] and were used as “donor” cells in allogeneic trogocytosis assays. Similarly, transfected KG-1 cells (KG1-HLA-G1), U937 cells (U937-HLA-G1), and THP-1 cells (THP-1-HLA-G1) were used as membrane “donor cells” in autologous trogocytic assays. NKL cells were maintained in medium supplemented with 10 IU/ml of IL-2 (Sigma), whereas U937, THP-1, HL-60, KG-1, LCL, Ramos, Raji, RPMI8226, U266, and Jurkat cell lines were not. Culture medium was RPMI 1640 (Invitrogen) supplemented with 2 mM l-glutamine, 1 μg/ml of gentamicin and fungizone (Sigma), and 10% of heat-inactivated FCS (Invitrogen).

### Antibodies and flow cytometry

PC5-conjugated anti-CD19 and anti-CD5 were from Miltenyi; PE-conjugated anti-HLA-G1 MEM-G/9 was obtained from Exbio, Praha; and PE-conjugated anti-CD3 was from Beckman Coulter. Biotin-coupled anti-CD4 and PC5-conjugated anti-biotin antibody were from Miltenyi. Purified PC5- and PE-conjugated isotype controls were from Miltenyi. For flow-cytometry analyses, Fc receptors were blocked by a 30-min incubation with 1 μg/μl of pooled purified isotype antibodies in PBS_1x_. All staining steps were performed on ice or at less than 4°C and isotype-matched control antibodies were systematically used. Flow-cytometry analyses were performed on a Canto II cytometer (Beckton Dickinson) using FlowJo software (Tree Star).

### Trogocytosis assays

#### Trogocytosis assays between allogeneic tumor cells

Thirty-minute co-incubations were set-up between “acceptor cells” (cell lines or *ex vivo* B-CLL, B lymphoma, and T lymphoma cells) and LCL-HLA-G1 “donor” cells whose membranes had been pre-labeled with the lipophilic dye PKH67 (Sigma) following the manufacturer’s recommendations. We used a 1:1 donor-acceptor ratio, a total concentration of 10^6^ to 10^7^ cells/ml, and incubation at 37°C in a 5% CO_2_-humidified incubator. At the end of the co-incubation, the cells were placed on ice and all further steps were performed at less than 4°C. Acquisition of donor cell-derived membrane and HLA-G1 by acceptor cells was investigated by flow cytometry.

#### Trogocytosis assays between cells from the same tumor cell line

To evidence trogocytosis capabilities in autologous conditions, tumor line cells were split into PKH67-labeled “donor” cells and PKH67-negative “acceptor” cells, and then co-incubated back together for 30 min at a 1:1 donor-acceptor ratio (total concentration of 10^6^ to 10^7^ cells/ml), and at 37°C in a 5% CO_2_ humidified incubator. The transfer of donor PKH67-labeled membranes onto “acceptor” trogocytic cells was analyzed by flow cytometry. To evidence trogocytic transfer of HLA-G between autologous tumor lines, HLA-G1-transfected cells were labeled with PKH67, and then incubated with their non-transfected counterparts in the same conditions as above. Acquisition of donor cell-derived membrane and HLA-G1 by acceptor cells was investigated by flow cytometry.

#### Trogocytosis assays between autologous PBMCs from patients with hematological tumors

Prior to trogocytosis assay, PBMCs were split. One half of the cells (“acceptor” cells) was labeled with PC5-conjugated anti-CD5 antibody (B-CLL patients), or CD4 (T cell lymphoma) (Miltenyi) and the other half (“donor” cells) was labeled with PKH67 (Sigma). Both pre-labeled cell populations were then co-incubated back together for 30 min at a 1:1 donor-acceptor ratio (total concentration of 10^6^ to 10^7^ cells/ml), and at 37°C in a 5% CO_2_-humidified incubator. The acquisition of PKH67-labeled membranes from donor cells by antibody pre-labeled acceptor cells was then analyzed by flow cytometry.

## Results

### Trogocytic capabilities of tumor cell lines *in vitro*

**Figure 1:**
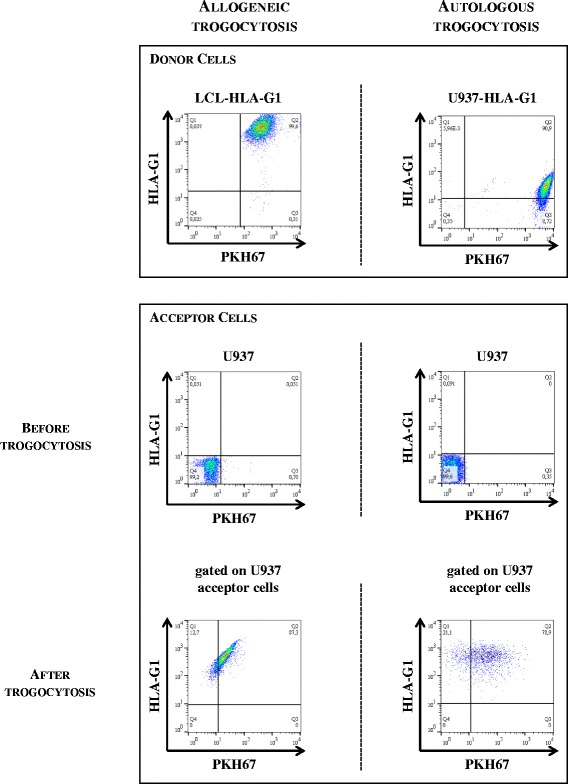
**Trogocytic capabilities of tumor cell lines** ***in vitro*** **.** As a representative example, trogocytosis capability results obtained for the U937 cell line are presented. Acceptor cells were U937 cells. Membrane-donor cells were LCL-HLA-G1 cells for allogeneic trogocytosis experiments, and U937-HLA-G1 for autologous trogocytosis experiments. Membrane-donor cells were labeled with the lipophilic dye PKH67 prior to trogocytosis assay. For both allogeneic and autologous trogocytosis experiments, membrane donor and membrane acceptor cells are shown prior to and at the end of the 30-min trogocytosis assay. After the trogocytosis assay, PKH67^hi^ donor cells were gated out and trogocytic PKH67^low^ cells and HLA-G1^low^ cells can be seen. Results shown are representative of three independent experiments.

We performed experiments aimed at demonstrating that tumor cell lines were trogocytic and capable of acquiring membranes and the membrane-bound molecule HLA-G from other cells, be they allogeneic or autologous. In these experiments, all membrane acceptor cells were HLA-G-negative, and all membrane-donor cells had been pre-labeled with the membrane dye PKH67. For allogeneic trogocytosis experiments, membrane-donor cells were LCL-HLA-G1 cells since previous work had demonstrated that they constituted good membrane and HLA-G1 donor cells [40,46]. For autologous trogocytosis experiments, membrane-donor cells were cells from the same cultures as the acceptor cells, which had been pre-labeled with PKH67, or, when available, PKH67-labeled HLA-G1-transfected counterparts of the HLA-G1-negative acceptor cells. After the 30-min co-incubations of donor and acceptor cells, the transfers of membrane and HLA-G1 onto the acceptor cells were evaluated by flow cytometry using PKH67-associated and anti-HLA-G antibody-associated fluorescence. As an illustration, the results obtained for the U937 cell line are shown in Figure 1. As can be seen, on the left for allogeneic trogocytosis experiments and on the right for autologous trogocytic experiments, donor cells (LCL-HLA-G1 and U937-HLA-G1, respectively) were double-positive for PKH67-associated fluorescence and membrane-bound HLA-G1 (PKH67^hi^HLA-G1^hi^ donor cells). Prior to trogocytosis experiments, acceptor U937 cells were PKH67 and HLA-G1 double-negative. At the end of the experiments, donor cells were still-identified as PKH67^hi^HLA-G1^hi^ cells and were gated out. Figure 1 shows that after allogeneic trogocytosis assay, 87.3% of the originally PKH67- and HLA-G1-negative acceptor U937 cells displayed PKH67-associated fluorescence, and 100% displayed HLA-G1-associated fluorescence. Similarly, after autologous trogocytosis assay, 78.9% of the originally PKH67- and HLA-G1-negative acceptor U937 cells now display PKH67-associated fluorescence, and 100% display HLA-G1-associated fluorescence. These results indicate that U937 acceptor cells have the capability to acquire membranes and membrane-bound HLA-G1 from allogeneic LCL-HLA-G1 cells and also from autologous, HLA-G1-expressing U937 cells.

The results obtained for all cell lines are presented in Table 1. They show that all cell lines studied were trogocytic, but differed in the extent of their capability to acquire membranes and membrane-associated molecules from allogeneic and autologous cells. Indeed, the proportion of acceptor cells that had acquired membranes and/or membrane-bound HLA-G from LCL-HLA-G1 cells ranged from 14% (NKL cells) to 99.8% (U937 cells) in allogeneic conditions, and from 35% (KG-1 cells) to 100% (U937 cells) in autologous conditions. The fact that these cell lines were trogocytic in both conditions may indicate that the mechanisms that underlie trogocytosis transfers might not differ between allogeneic and autologous situations. No obvious lineage-related differences were observed, and within one lineage, cell lines differed in their trogocytic capabilities. U937 and Ramos cells were the most trogocytic among the myeloid and B cell lineages, respectively. The differences between trogocytic capabilities of cell lines of the same lineage might be due to long-term cell culture, and also to the tumor’s differentiation stage. Finally, HLA-G1 transfer could systematically be observed. Overall, except for the U937 and Ramos cell lines which displayed consistent high trogocytic capabilities, the inter-experimental variability was significant (Additional file 1: Table S1), owing to factors that are obviously related to cell culture. Because the mechanism of trogocytosis between tumor cells is not known, we could not identify these factors.

**Table 1:** **Trogocytic capabilities of tumor cell lines in vitro**

		**Allogeneic trogocytic capability**		**Autologous trogocytic capability**			**Autologous (** ***n*** **)**
	**Cell line**	**% of membrane acquired**	**% of HLA-G1 acquired**	**% of membrane acquired**	**% of HLA-G1 acquired**		
**Monocytic**	U937	62.9	99.8	41.6	100.0	3	3
	THP-1	18.2	36.7	36	3.7	3	3
	HL-60	23.3	30.8	53.2	n/a	3	3
	KG1	22.5	50.4	35.5	36.5	3	3
**B cells**	Ramos	73.5	77.9	37.1	n/a	3	2
	Raji	19.8	25.6	54.2	n/a	3	3
	RPMI 8226	25.5	23.8	84.8	n/a	3	3
	U266	20.2	30.1	75.2	n/a	3	3
**T cells**	Jurkat	16.4	35.9	35.6	n/a	3	3
**NK cells**	NKL	14.2	37.9	47.9	n/a	3	3

The capabilities of tumor cell lines to acquire membranes and membrane-associated HLA-G were investigated using PKH67-labeled allogeneic (LCL-HLA-G1) and PKH67-labeled autologous cells. For each experiment type, acceptor cells, donor cells, and the mean percentage of acceptor cells that acquired membranes (PKH67) and/or HLA-G1 from donor cells is shown. The number of independent experiments performed for each line is shown. *n*/*a* not applicable for lack of HLA-G-positive autologous lines.

### Trogocytic capabilities of tumor cells from hematological malignancies

We next investigated if the previous results obtained *in vitro* using tumor cell lines held true for human tumors *in vivo*. Because B cells and B cell lines were shown to have a high trogocytic capability [50], we focused primarily on B cell tumors, but 2 T cell lymphomas were also included. Because *bona fide in vivo* evaluation of membrane exchanges between cells of the same liquid tumor is not possible, we performed *ex vivo* experiments, keeping to an absolute minimum the experimental time and cell manipulation (see experimental procedures): experiments lasted less than 2 h altogether from the time of blood collection to the end of the trogocytosis assays.

As for cell lines above, we evaluated the trogocytic capability of hematological tumor cells to acquire membranes and membrane-bound HLA-G1 from allogeneic LCL-HLA-G1 cells, and to exchange membranes among each other in an autologous fashion. The autologous exchange of membrane-bound HLA-G1 between cells of the same tumor could not be investigated for lack of HLA-G expression at the surface of the tumors we obtained.

For allogeneic trogocytosis experiments, membrane-donor cells were PKH67-labeled LCL-HLA-G1 cells and acceptor cells were either CD19^+^ (B-CLL and B cell lymphomas) or CD3ˉCD4^+^ (T cell lymphomas) PKH-negative tumor cells.

**Figure 2:**
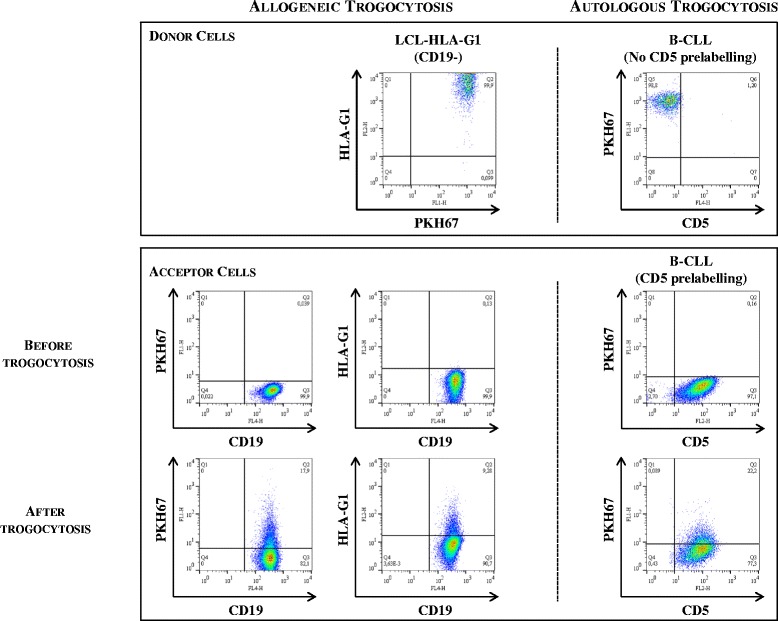
**Trogocytosis capabilities of freshly isolated hematological tumor cells** ***ex vivo*** **.** As a representative example, trogocytosis capability results obtained for tumor cells from the patient B-CLL 15 are presented. In allogeneic trogocytosis assays, donor cells were PKH67-prelabeled LCL-HLA-G1 cells and acceptor cells were CD19^+^ B-CLL cells. In autologous trogocytosis assays, donor and acceptor cells were from the same blood sample; donor cells were PKH67-prelabeled and acceptor cells were prelabeled with CD5. For both allogeneic and autologous trogocytosis experiments, membrane donor and membrane acceptor cells are shown prior to and at the end of the 30-min trogocytosis assay. After the trogocytosis assay, trogocytic, PKH67^hi^ donor cells were gated out, and PKH67^low^ cells and HLA-G1^low^ acceptor B-CLL cells can be seen.

As an illustration, the results obtained for cells from one representative B-CLL patient (patient B-CLL 15) are shown in Figure 2. As can be seen, on the left for allogeneic trogocytosis experiments and on the right for autologous trogocytic experiments, donor cells (CD19ˉ LCL-HLA-G1 and CD19^+^-gated B-CLL tumor cells, respectively) were positive for PKH67-associated fluorescence and, in the case of LCL-HLA-G1 cells, for membrane-bound HLA-G1 as well (PKH67^hi^ donor cells). Acceptor cells in allogeneic trogocytosis experiment were CD19^+^ B-CLL tumor cells and were negative for PKH67-associated fluorescence and HLA-G1. In autologous trogocytosis experiments, in order to distinguish donor and acceptor cells, acceptor cells were pre-labeled with CD5 prior to their use. In our displays, CD5 specifically marks acceptor cells even though donor and acceptor cells express it because this molecule does not transfer from cell to cell by trogocytosis (Additional file 2: Figure S1). At the end of the experiments, donor cells were still identified as PKH67^hi^ cells and were gated out, non-trogocytic acceptor cells were still PKH67-negative, whereas trogocytic cells were characterized by low PKH67 levels. As can be observed for allogeneic trogocytosis, 17.9% of the originally PKH67- and HLA-G1-negative CD19^+^-gated acceptor B-CLL cells displayed PKH67-associated fluorescence, and 9.3% display HLA-G1-associated fluorescence, indicating that B-CLL tumor cells are trogocytic and can acquire membranes and membrane-bound HLA-G1 from allogeneic cells. In autologous trogocytosis, 22.2% of the originally PKH67- and HLA-G1-negative CD19^+^-gated acceptor B-CLL cells displayed PKH67-associated fluorescence, demonstrating that B-CLL tumor cells can acquire membranes from autologous cells.

Five such experiments could be performed, three on B-CLL, one on a B cell lymphoma, and one on T cell lymphoma. In the latter case, acceptor tumor T cells were CD3^−^CD4^+^ and were distinguished from donor T cells by CD4 pre-labeling since CD4 does not transfer by trogocytosis [40]. In all cases, trogocytosis was observed between donor and acceptor cells from the same tumor, with transfer extents varying between 8.1% and 55.7% of the acceptor tumor cell population. The results obtained for each individual tumor are shown in Table 2. Interestingly, these experiments also showed the very low trogocytic capabilities of acceptor CD3^+^CD5^+^ T cells in autologous conditions (Additional file 3: Figure S2). This is similar to what had been demonstrated in the context of multiple myeloma [51]. Thus, these experiments demonstrate that in the pathological context studied, trogocytosis essentially occurs between hematopoietic tumor cells *ex vivo*.

**Table 2:** **Trogocytosis capabilities of tumor cells from hematological malignancy patients**

	**Allogeneic trogocytosis**			**Autologous trogocytosis**	
	**Donor cells**	**Trogocytic cells (%)**		**Donor cells**	**Trogocytic cells (%)**
**Acceptor cells**		**PKH67**	**HLA-G1**		**PKH67**
**T lymphoma**	**LCL-HLA-G1**	57.8	35.5	**T lymphoma**	55.7
**B cell Lymphoma (mantle)**		12.6	12.5	**B cell lymphoma (mantle)**	8.1
**B-CLL 15**		17.9	9.3	**B-CLL 15**	23
**B-CLL 14**		14.7	11.7	**B-CLL 14**	11.5
**B-CLL 13**		15	9.1	**B-CLL 13**	22.2
**B-CLL 12**		0.8	15.7		
**B-CLL 11**		2.5	19.1		
**B-CLL 10**		nt	23.4		
**B-CLL 9**		nt	30.4		
**B-CLL 8**		nt	16.7		
**B-CLL 7**		nt	29		
**B-CLL 6**		nt	30.1		
**B-CLL 5**		nt	5		
**B-CLL 4**		nt	28.5		
**B-CLL 3**		nt	9.6		
**B-CLL 2**		nt	7.4		
**B-CLL 1**		nt	21		

Experiments were performed as described in materials and methods and as exemplified in Figure 2 for patient B-CLL 15. The individual results obtained for each indicated patient are presented. For both allogeneic and autologous trogocytosis experiments, acceptor and donor cells are indicated. The percentage of trogocytic cells is the percentage of acceptor cells that acquired PKH67 and/or HLA-G1 from PKH67^hi^ donor cells. *nt* not tested.

## Discussion

Trogocytosis, i.e., the capability to acquire membranes and membrane-associated molecules from a cell by another, has been described for non-tumor immune cells and shown to impact their immunological functions. In this work, we investigated whether tumor cells of immune origin were also trogocytic. Working with hematological cell lines and freshly isolated hematological tumors, we demonstrate here that trogocytic function was indeed preserved in hematological tumors, that membrane exchanges happen in autologous situations, and hence are likely to occur *in vivo*.

Our previous studies on trogocytosis between tumor cell lines and non-tumoral immune cells showed that effector T and NK cells were capable of acquiring membranes and HLA-G1 protein from HLA-G1-expressing tumor cell lines, and that trogocytosis or HLA-G1 acquisition was not dependent on HLA-G1. Indeed, blocking the HLA-G1/ILT2 interaction did not prevent HLA-G1 from being transferred from tumor to immune cells. Rather, HLA-G1 transferred because it was part of transferred membrane patches whose trogocytosis was dependent on other molecules that could not be identified, even though CD28/CD86 or MHC-TCR interactions were ruled out [40]. Fixation of the donor cell’s membrane using PFA was previously shown to inhibit HLA-G1 trogocytic transfer from tumor cells to activated T cells [40], and this was confirmed here for tumor-to-tumor transfers (Additional file 4: Figure S3).

One of the most striking features of trogocytic transfers is that the acceptor cells may display membrane proteins that they do not express themselves. This has been demonstrated for T and NK cells in the context of HLA-G1 trogocytosis [22,40]. As a consequence, the acquired proteins (e.g., HLA-G1) are displayed on the acceptor cells for a limited time only. Previous studies from our laboratory showed that HLA-G1 that had been acquired by trogocytosis was no longer present at the cell-surface after 24 h for T and NK acceptor cells, and 6 h for monocytes [22,40,47]. Experiments performed in the context of the present study confirmed that the life time of acquired HLA-G at the surface of acceptor tumor cells is limited, and of the order of 24 h (data not shown).

We previously reported that HLA-G does not direct its own transfer, and seems to transfer passively from cell to cell. This relates to the fact that many proteins are transferred during trogocytosis, including costimulatory molecules such as CD86 or CD54, receptors such as ILT2, and MHC class I and class II molecules [40]. Many more have not been investigated, and it is likely that most of them also transfer passively. This does not mean that any molecule can transfer by trogocytosis. Indeed, we reported that ILT3 or CD4, CD8, CD14, CD19, and here CD5 are not concerned by trogocytosis, which suggests that trogocytic exchanges are not random processes, but regulated ones.

This begs the question of what the molecules that mediate trogocytic transfers are. Previous work has focused on trogocytosis between antigen-presenting cells and T cells, or between target cells and cytotoxic T or NK cells. In most of these cases, trogocytic transfers were associated with antigen-specificity (T cells) or function (i.e., target recognition, NK cells) [22,52]. However, previous studies show that trogocytic transfers are only partially dependent on antigen-specificity: for instance, in case of antigen-specific interaction between non-activated donor and acceptor T cells, the TCR is involved in the transfer process, but when T lymphocytes are activated, trogocytosis occurs independently of TCR engagement, and with a higher efficiency [40]. In another study, we demonstrated trogocytosis between autologous cells in the absence of foreign antigen [43], and in the present report, we show that B and monocytic tumor cell lines are capable of trogocytosis in autologous conditions and in the absence of antigen, strengthening the notion that trogocytosis is at least partially independent of antigen-specificity. Nonetheless, the molecules that are responsible for trogocytosis remain elusive.

B-CLL progressively lost their trogocytic capability upon culture, sometimes as fast as in 24 h (data not shown). This indicates that the trogocytic capability of tumor cells is highly dependent on microenvironmental factors, which means that the levels of trogocytosis might be higher *in vivo* than what we observed *ex vivo*. As stated above, we do not know which molecules may be responsible for tumor-to-tumor membrane exchanges. It is therefore difficult to predict which microenvironment factors may modulate the expression and/or function of these molecules and, down the line, the extent of trogocytic exchanges. Keeping B-CLL in culture medium supplemented with autologous serum rather than FCS allowed B-CLL cells to retain their trogocytic capabilities longer (not shown). Thus, microenvironmental factors supporting trogocytic capability might be cytokines, growth factors, stress factors, and more generally molecules present within the serum, rather than nutrient deprivation or microenvironmental conditions such as hypoxia for instance.

Trogocytic exchanges between tumor cells *in vivo* are relevant because we demonstrated that the transferred proteins might be functional in their new cell hosts. This is even more likely if donor and acceptor cells are cells of the same tumor. We already demonstrated *in vitro* that trogocytosis could contribute to tumor escape. Indeed, HLA-G1 acquisition by T cells from HLA-G1-expressing tumor cells rendered them temporarily suppressive. Recently, this hypothesis was strengthened by Brown et al. [51], who confirmed our previous *in vitro* results and demonstrated in an all-autologous *ex vivo* system that T cells can acquire HLA-G from autologous multiple myeloma tumor cells, turning them into regulatory cells. Here, we also show that T cells can acquire membranes from autologous B-CLL *ex vivo*, and confirm that such transfers remain rare events. However, our results, obtained with the same blood samples, show that in B-CLL, tumor-to-tumor membrane exchanges are prominent. The meanings of such exchanges remain to be explored in depth, but already the interest for the tumor of transferring HLA-G between its constituent cells can be envisioned. For instance, it is known that some but not all B leukemia cells and multiple myeloma cells from the same tumor can express HLA-G [38,53-56], which is capable of protecting them from NK cell cytolysis, and thus constitutes an immune escape mechanism [57]. Therefore, by transferring HLA-G and possibly other immune-inhibitory molecules, tumor cells may share some of their immune escape strategies, as our *in vitro* model showed here. For a tumor whose cellular constituents heterogeneously express immune escape molecules such as HLA-G, trogocytic sharing presents definite advantages, such as (i) conferring extra-protection to those cells which do not express the transferred protein, and (ii) diversifying the array of immune escape strategies that each cell uses. This would strengthen the overall resistance of the whole tumor to anti-tumoral immunity. This also means that blocking trogocytosis might constitute a new way to restore anti-tumor immunity, and emphasizes the need to precisely characterize the molecular mechanisms that are involved in trogocytic exchanges.

## Additional files

Additional file 1: Table S1. Inter-experimental variability for trogocytic capabilities of tumor cell lines in vitro. Standard deviation values (STD) obtained for the data presented in Table I. Additional file 2: Figure S1. CD5 does not transfer to acceptor cells by trogocytosis. The representative results obtained for patient number B-CLL 15 are shown. Freshly isolated PBMC from B-CLL patients were split and labeled or not with anti-CD5, generating donor and acceptor cell populations, respectively. Donor and acceptor cells were then used in a trogocytosis assay and the transfer of CD5 from the CD5^hi^ donor cells to acceptor CD19^+^ cells was investigated by flow cytometry. No transfer of CD5 was observed in any patient, and after trogocytosis assay, CD5^+^ and CD5- populations were still clearly distinguishable. Additional file 3: Figure S2. Low trogocytic capabilities of CD3+ T cells from B**-**CLL patients. Autologous trogocytosis experiments were performed as detailed in the text and PKH67 acquisition by CD5-prelabelled acceptor CD3^+^ T cells was studied. Representative results obtained for patient B-CLL 15 are shown. PKH acquisition by acceptor B-CLL cells (22%) and by CD3^+^ T cells (3.5%) are shown. Additional file 4: Figure S3. Fixation of donor cells with PFA prevents trogocytosis. Representative results obtained for one B-CLL patient are shown. Autologous trogocytosis experiments were performed as described in Materials and Methods, except that donor cells were either fixed with 0.5% paraformaldehyde in PBS_1x_ (PFA) or not prior to the trogocytosis assay. The transfer of PKH67-labeled membranes onto acceptor cells was investigated by flow cytometry. No trogocytic transfer was observed when fixed donor cells were used.
