# Supplementary material for: Trogocytic intercellular membrane exchanges among hematological tumors
Source: J Hematol Oncol. 2015 Mar 14;8:24. doi: 10.1186/s13045-015-0114-8 (PMC4371622; doi:10.1186/s13045-015-0114-8)
Supplement: Additional file 1: Table S1. — Inter-experimental variability for trogocytic capabilities of tumor cell lines in vitro. Standard deviation values (STD) obtained for the data presented in Table I. [file 13045_2015_114_MOESM1_ESM.doc]

Additional file 1

Inter-experimental variability for trogocytic capabilities of tumor cell lines in vitro

|  |  | Allogeneic trogocytic experiments | | Autologous trogocytic experiments | | Allogeneic (n) | Autologous (n) |
| --- | --- | --- | --- | --- | --- | --- | --- |
|  | Cell line | STD membrane acquired | STD HLA-G1 acquired | STD membrane acquired | STD HLA-G1 acquired |  |
| Monocytic | U937 | 29,7 | 0,4 | 52,8 | 0,0 | 3 | 3 |
|  | THP-1 | 15,2 | 14,0 | 9,8 | 2,6 | 3 | 3 |
|  | HL-60 | 17,8 | 14,2 | 3,7 | n/a | 3 | 3 |
|  | KG1 | 32,9 | 41,8 | 24,0 | 32,6 | 3 | 3 |
| B cells | Ramos | 7,4 | 18,4 | 15,8 | n/a | 3 | 2 |
|  | Raji | 5,3 | 4,5 | 19,4 | n/a | 3 | 3 |
|  | RPMI 8226 | 30,0 | 16,1 | 25,9 | n/a | 3 | 3 |
|  | U266 | 18,4 | 25,7 | 40,1 | n/a | 3 | 3 |
| T cells | Jurkat | 10,1 | 10,9 | 24,6 | n/a | 3 | 3 |
| NK cells | NKL | 13,4 | 12,6 | 25,6 | n/a | 3 | 3 |
