# Supplementary material for: Trogocytic intercellular membrane exchanges among hematological tumors
Source: J Hematol Oncol. 2015 Mar 14;8:24. doi: 10.1186/s13045-015-0114-8 (PMC4371622; doi:10.1186/s13045-015-0114-8)
Supplement: Additional file 2: Figure S1. — CD5 does not transfer to acceptor cells by trogocytosis. The representative results obtained for patient number B-CLL 15 are shown. Freshly isolated PBMC from B-CLL patients were split and labeled or not with anti-CD5, generating donor and acceptor cell populations, respectively. Donor and acceptor cells were then used in a trogocytosis assay and the transfer of CD5 from the CD5hi donor cells to acceptor CD19+ cells was investigated by flow cytometry. No transfer of CD5 was observed in any patient, and after trogocytosis assay, CD5+ and CD5- populations were still clearly distinguishable. [file 13045_2015_114_MOESM2_ESM.pptx]

## Slide 1
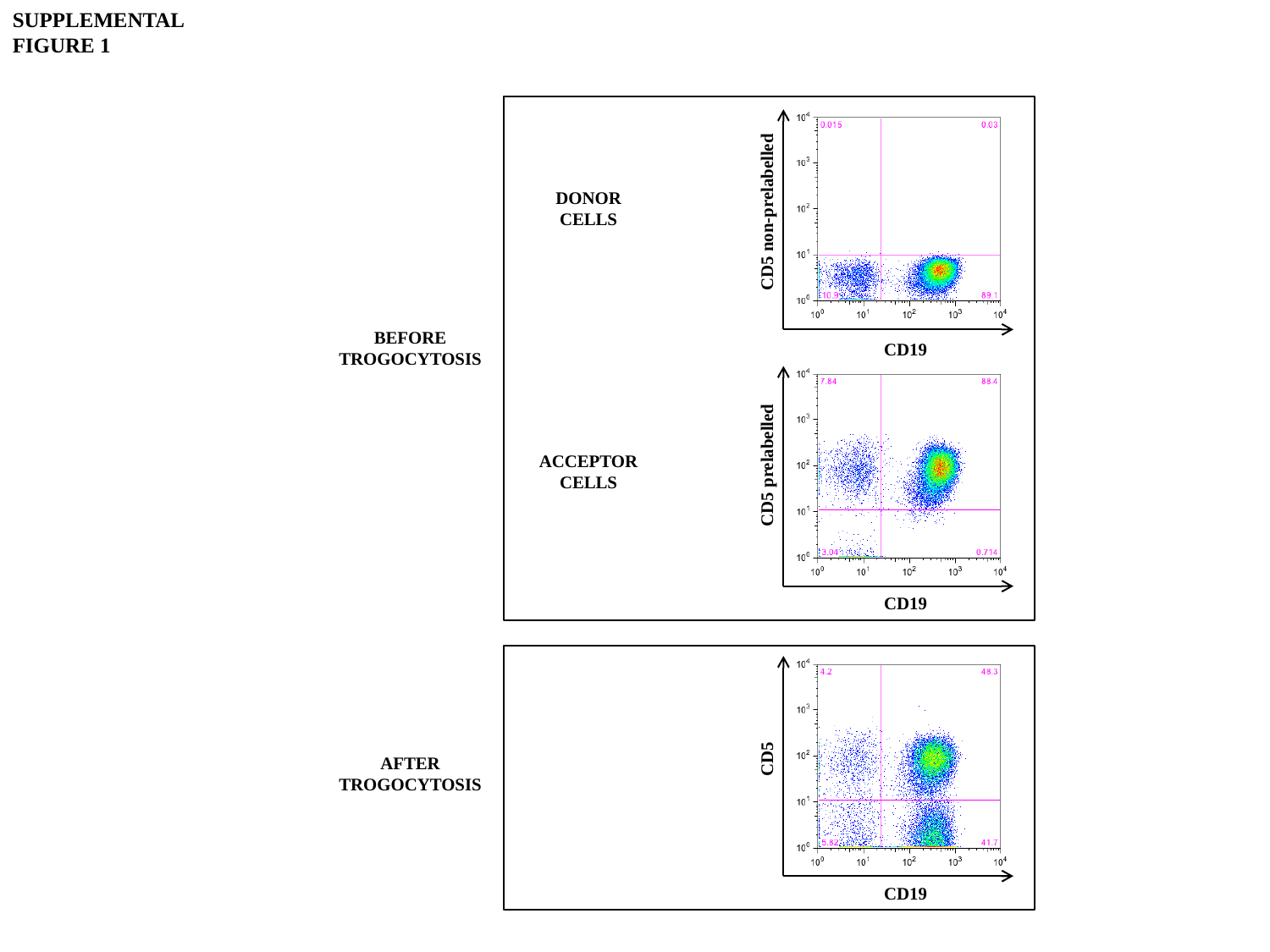

Supplemental Figure 1
Donor cells
CD5 non-prelabelled
Before Trogocytosis
CD19
Acceptor cells
CD5 prelabelled
CD19
CD5
After Trogocytosis
CD19
