# Supplementary material for: Trogocytic intercellular membrane exchanges among hematological tumors
Source: J Hematol Oncol. 2015 Mar 14;8:24. doi: 10.1186/s13045-015-0114-8 (PMC4371622; doi:10.1186/s13045-015-0114-8)
Supplement: Additional file 3: Figure S2. — Low trogocytic capabilities of CD3+ T cells from B-CLL patients. Autologous trogocytosis experiments were performed as detailed in the text and PKH67 acquisition by CD5-prelabelled acceptor CD3+ T cells was studied. Representative results obtained for patient B-CLL 15 are shown. PKH acquisition by acceptor B-CLL cells (22%) and by CD3+ T cells (3.5%) are shown. [file 13045_2015_114_MOESM3_ESM.pptx]

## Slide 1
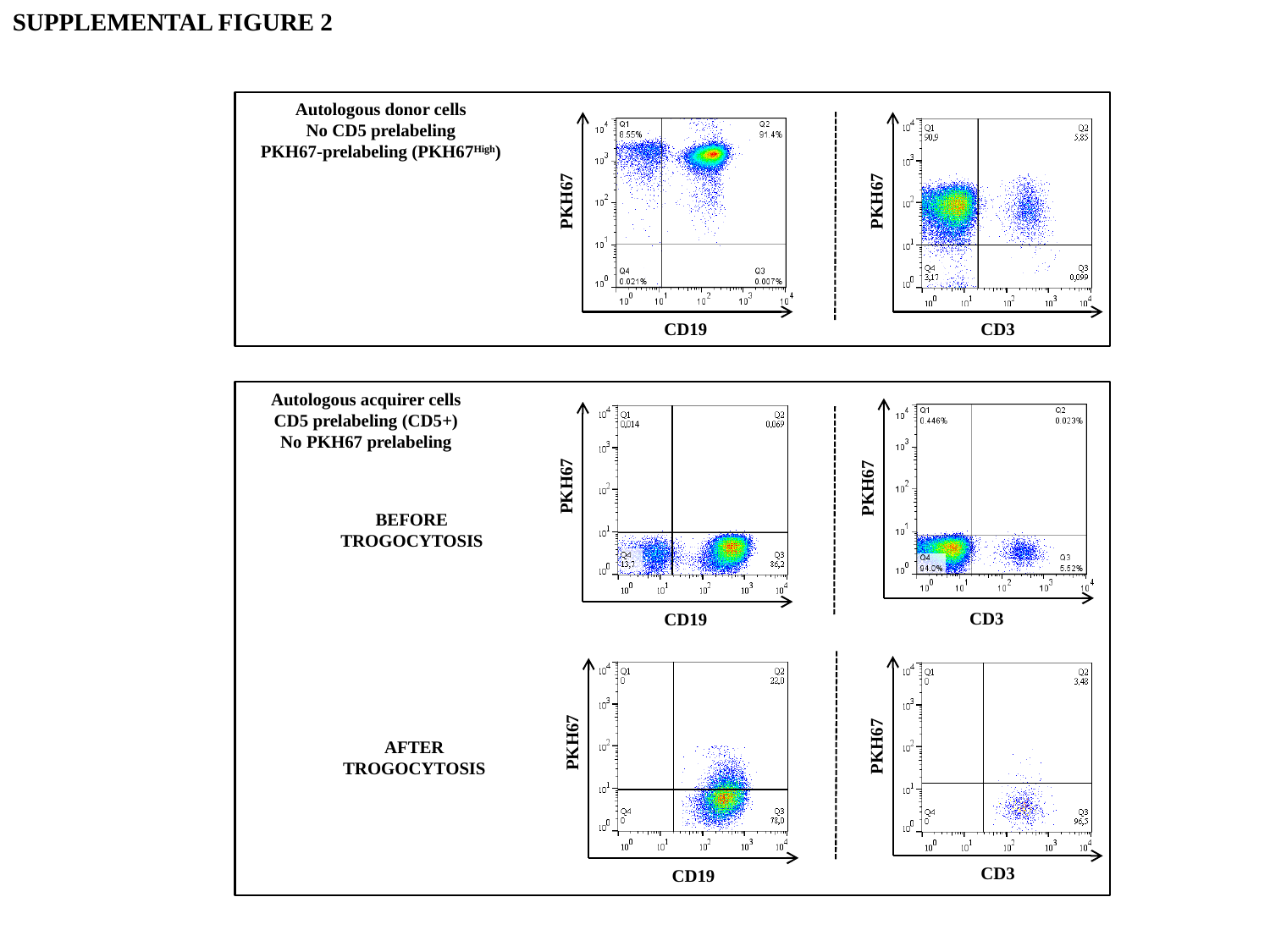

Supplemental Figure 2
Autologous donor cells
No CD5 prelabeling
PKH67-prelabeling (PKH67High)
PKH67
PKH67
CD19
CD3
Autologous acquirer cells
CD5 prelabeling (CD5+)
No PKH67 prelabeling
PKH67
PKH67
Before trogocytosis
CD3
CD19
PKH67
After trogocytosis
PKH67
CD3
CD19
