# Supplementary material for: Trogocytic intercellular membrane exchanges among hematological tumors
Source: J Hematol Oncol. 2015 Mar 14;8:24. doi: 10.1186/s13045-015-0114-8 (PMC4371622; doi:10.1186/s13045-015-0114-8)
Supplement: Additional file 4: Figure S3. — Fixation of donor cells with PFA prevents trogocytosis. Representative results obtained for one B-CLL patient are shown. Autologous trogocytosis experiments were performed as described in Materials and Methods, except that donor cells were either fixed with 0.5% paraformaldehyde in PBS1x (PFA) or not prior to the trogocytosis assay. The transfer of PKH67-labeled membranes onto acceptor cells was investigated by flow cytometry. No trogocytic transfer was observed when fixed donor cells were used. [file 13045_2015_114_MOESM4_ESM.pptx]

## Slide 1
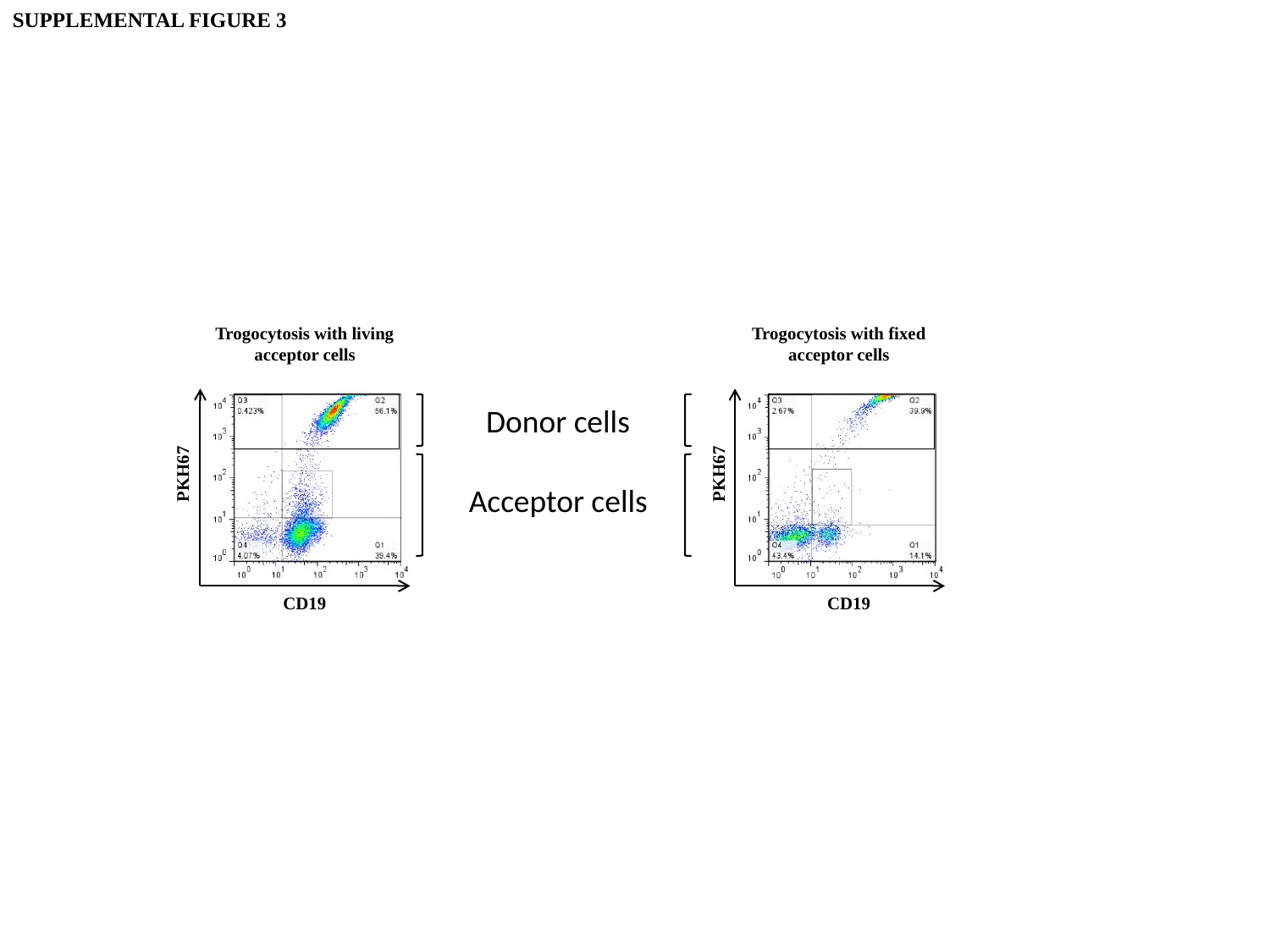

Supplemental Figure 3
Trogocytosis with fixed acceptor cells
Trogocytosis with living acceptor cells
Donor cells
PKH67
PKH67
Acceptor cells
CD19
CD19
